# Major and Trace Airborne Elements and Ecological Risk Assessment: Georgia Moss Survey 2019–2023

**DOI:** 10.3390/plants13233298

**Published:** 2024-11-23

**Authors:** Omari Chaligava, Inga Zinicovscaia, Alexandra Peshkova, Nikita Yushin, Marina Frontasyeva, Konstantin Vergel, Makhabbat Nurkassimova, Liliana Cepoi

**Affiliations:** 1 Joint Institute for Nuclear Research, 6 Joliot-Curie Str., 141980 Dubna, Russia; zinikovskaia@mail.ru (I.Z.); peshkova.alexandra92@gmail.com (A.P.); ynik_62@mail.ru (N.Y.); marina@nf.jinr.ru (M.F.); verkn@mail.ru (K.V.); 2 Doctoral School of Natural Sciences, Moldova State University, 75A M. Kogalniceanu Str., MD-2009 Chisinau, Moldova; liliana.cepoi@imb.utm.md; 3 Faculty of Informatics and Control Systems, Georgian Technical University, 77 Merab Kostava Str., 0171 Tbilisi, Georgia; 4 Horia Hulubei National Institute for R&D in Physics and Nuclear Engineering, 30 Reactorului Str., 077125 Magurele, Romania; 5 Faculty of Natural Sciences, L.N. Gumilyov Eurasian National University, 2 Satpayev Str., 010008 Astana, Kazakhstan; maha.bilan@mail.ru; 6 Institute of Microbiology and Biotechnology, Technical University of Moldova, 1 Academiei Str., MD-2028 Chisinau, Moldova

**Keywords:** Georgia, air pollution, trace elements, moss biomonitoring, ICP-AES, DMA-80 Milestone

## Abstract

The study, carried out as part of the International Cooperative Program on Effects of Air Pollution on Natural Vegetation and Crops, involved collecting 95 moss samples across the territory of Georgia during the period from 2019 to 2023. Primarily samples of *Hypnum cupressiforme* were selected, with supplementary samples of *Abietinella abietina*, *Pleurozium schreberi*, and *Hylocomium splendens* in cases of the former’s absence. The content of 14 elements (Al, Ba, Cd, Co, Cr, Cu, Fe, Mn, Ni, Pb, S, Sr, V, and Zn) was detected using Inductively Coupled Plasma Atomic Emission Spectroscopy (ICP-AES), while the Hg content was determined using a Direct Mercury Analyzer. To identify any relationships between chemical elements and to depict their sources, multivariate statistics was applied. Principal component analysis identified three main components: PC1 (geogenic, 43.4%), PC2 (anthropogenic, 13.3%), and PC3 (local anomalies, 8.5%). The results were compared with the first moss survey conducted in Georgia in the period from 2014 to 2017, offering insights into temporal trends of air quality. Utilizing GIS, a spatial map illustrating pollution levels across Georgia, based on the Pollution Load Index, was generated. The Potential Environmental Risk Index emphasized significant risks associated with mercury and cadmium at several locations. The study highlights the utility of moss biomonitoring in assessing air pollution and identifying hotspots of contamination. The findings from this study could be beneficial for future biomonitoring research in areas with varying physical and geographical conditions.

## 1. Introduction

Air pollution, originating from both human activities and natural sources, is one of the major challenges facing the world [1]. The connection between air pollution, primarily sourced from activities such as power generation, waste incineration, industrial processes, transport, etc. and the occurrence of diseases is becoming increasingly evident [2,3].

The impact of air pollution on human health can be categorized into short-term and long-term [4,5]. Short-term effects include temporary illnesses such as pneumonia or bronchitis, along with discomfort like irritation to the nose, throat, eyes, or skin. Long-term effects can last for years or even a lifetime, leading to conditions like heart disease, lung cancer, and respiratory diseases. In extreme cases, air pollution can cause birth defects and even death [6,7].

One of the most significant components of air pollution is particulate matter (PM). PM specifically refers to tiny solid and liquid particles suspended in the air. The size and composition of PM adversely affects living organisms, ecosystems, and climate, consequently impacting the economy of the countries [8,9,10,11]. PM particles can contain a wide range of substances, including potentially toxic elements (PTEs) [12] such as As, Cd, Cr, Hg, and Pb, which pose a significant ecological risk due to their resistance to biodegradation and chemical breakdown [13]. This resistance allows them to persist within the environment, thereby contributing to ecological imbalances, which can disrupt the natural equilibrium of ecosystems, leading to detrimental effects on biodiversity and the overall health of the environment and population [13].

The rising threat of air pollution and associated health risks have highlighted the need for the improvement of air quality monitoring. Monitoring the composition of the atmospheric deposition is a critical part of environmental protection as it helps in understanding the extent and nature of air pollution, allowing more effective control measures to be applied. Developing economies often lack the resources to investigate ambient air quality, resulting in insufficient monitoring and management of air pollution [14].

Moss biomonitoring is a cost-effective and versatile approach to monitor pollutants like trace elements, organic compounds, nitrogen, and microplastics across large areas, which makes it a suitable alternative to traditional monitoring techniques that are often expensive and limited by specific monitoring stations [15]. This method exploits the morphological and physiological properties of naturally occurring mosses, such as a wide geographical distribution, a large surface area, and the ability to obtain nutrients primarily through wet and dry deposition [16]. Mosses are particularly adept at retaining particles containing pollutants, and their concentrations can serve as a clear indicator of particle deposition patterns in different scenarios [15]. The simplicity of the moss collection process, coupled with the lack of requirement for expensive equipment for air and precipitation sampling, makes it a less laborious and more accessible method for assessing environmental pollution levels [17]. This technique, despite not providing direct quantitative measurements of deposition, can still offer valuable insights into atmospheric pollution levels by applying various mathematical approaches to analyze the elemental composition of moss samples [18,19].

The research was carried out as part of the International Cooperative Program on Effects of Air Pollution on Natural Vegetation and Crops (ICP Vegetation). This program operates under the Working Group on Effects (WGE) of the UNECE Convention on Long-Range Transboundary Air Pollution (LRTAP). The program’s research encompasses the effects of ozone pollution, the atmospheric deposition of heavy metals, nitrogen, and persistent organic pollutants (POPs) on vegetation. One of the key activities of ICP Vegetation is conducting moss surveys every 5 years to assess spatio-temporal changes in air quality [20]. The results of these surveys contribute to a broader understanding of air pollution distribution and its effects on ecosystems across Europe and beyond. The first moss survey in Georgia, conducted between 2014 and 2017, laid the groundwork for ongoing efforts to monitor and understand the impacts of air pollution on the country’s environment and population [21,22,23].

The objective of this research is to summarize the findings of the second moss survey conducted in Georgia, with the hypothesis that the content of major (Al, Fe, S) and trace elements in the moss samples will remain largely consistent with the findings of the previous survey, as there were no major changes in industrial activities, mining, transport or other sources of pollutant emissions in the area [24].

## 2. Results and Discussion

The descriptive statistics for 15 elements (Al, Ba, Cd, Cr, Co, Cu, Pb, Fe, Hg, Mn, Ni, S, Sr, V, and Zn) across 95 samples, including the range (minimum–maximum), arithmetic mean ± standard deviation (st.dev), median ± Median Absolute Deviation (MAD), coefficient of variation (CV%), quartiles (Q1 and Q3), 90th percentile, and calculated background levels are presented in Table 1. Descriptive statistics for each moss species are presented in Table S1. Each element’s content is given in milligrams per kilogram of dry weight. It is noted that all elements display non-normal distribution patterns and positive skewness. The highest coefficient of variation (CV%) is seen for Pb (94%), followed by Mn (74%), suggesting the presence of outliers within the dataset, which will be addressed subsequently in this paper. For the remaining elements CV% ranges from 26% to 53%.

The median values and range presented in Table 2 offer a rough comparison of the current findings with the locally conducted prior studies and an analogous study performed abroad [23,25,26,27]. Bulgaria serves as a relevant comparison for the Georgian biomonitoring results, sharing similarities with Georgia in terms of sampled moss species, latitude, and influence from the Black Sea. The median values of Co, Cr, Fe, Ni, Sr, and V, which are often of geological origin, are notably higher in Georgian samples relative to the Bulgarian ones. This suggests that the impact of soil composition, influenced by the geographical features of Georgia, namely slope erosion, is more pronounced. However, it is important to mention that concentrations of Al, Cd, Cu, and Zn are similar across both regions. It is suggested that in both countries vehicular emissions and the deposition of road dust constitute significant sources of atmospheric contamination.

While two neighboring countries in the Caucasus region exhibit mining and metallurgical operations as primary sources of emissions, notable differences in median concentrations of elements in mosses between Georgia and Armenia are revealed [26]. Several factors contribute to the lower elements concentrations in Georgia. One such factor is forest coverage. While forest lands occupy approximately 40.6% of Georgia’s territory, only about 11.1% of Armenia is forested [28,29,30]. This significant difference in land cover may amplify wind erosion effects in Armenia, resulting in higher elements concentrations in local mosses. Another factor influencing such differences is the type of moss species used. Most moss samples in the Armenia study were acrocarpous species, particularly *Syntrichia ruralis*. However, the manual recommends using pleurocarpus mosses [31]. This bias towards acrocarpous species may affect elemental uptake patterns.

A moss biomonitoring study using the moss *Hylocomium splendens* in Kazakhstan was performed close to large industrial centers and cities in the south of the country [27]. By comparing the results with Georgia, all element median values were much higher in Kazakhstan, except Cd, Cu, and Pb. Cadmium concentrations were nearly identical in terms of median values, but maximum values were higher in Kazakhstan. In the case of Pb, the median value was higher in Georgia, though concentration ranges were similar in both countries. The only element concentration that was lower in Kazakhstan was Cu. The differences in Cu concentration require further investigation.

Comparison of the results with the previous moss survey conducted in Georgia in 2014/2017 revealed a general trend of decreasing of the median values for the majority of the examined elements. However, notable exceptions were observed for Cd and Zn, whose median values remained unaltered throughout both survey periods. Conversely, Cu demonstrated an increase of median values during this timeframe. A Mann–Whitney U test showed that all the concentrations, except Cd, Pb, Zn, were significantly different (*p* < 0.05) from a previous survey [23].

It is important to highlight that out of the original sampling sites, only 33 remained unchanged or were within a 10 km radius of their previous positions, allowing collection of the same moss species as in the prior investigation [23]. The Mann–Whitney test applied to samples from these sites has also pinpointed considerable differences in the concentrations of all elements between moss samples collected in 2014/2017 and 2019/2023. Nevertheless, upon calculation of the Contamination Factors—calculated by dividing the measured concentration by the background level for each respective year—it became evident that there were minor, statistically insignificant variations observed for Pb and Cu only.

The variances observed between the moss surveys conducted in 2019/2023 and 2014/2017 could be attributed to alterations in the elements’ bioavailabilities, which were potentially influenced by the rates of wet and dry deposition during these years [32].

It is important to note that in the previous moss survey, neutron activation analysis (NAA) was used for the determination of the majority of the elements and atomic absorption spectrometry (AAS) was used specifically for Cd, Cu and Pb, whereas the current study used only ICP-AES. Given the non-destructive nature of NAA, it enables the quantification of the total amount of an element within a sample. In contrast, ICP-AES may not account for certain refractory compounds, whose presence can lead to incomplete digestion of samples, which may result in the omission of these compounds from the final analysis, affecting the accuracy and completeness of the elemental analysis [33].

**Figure 1:**
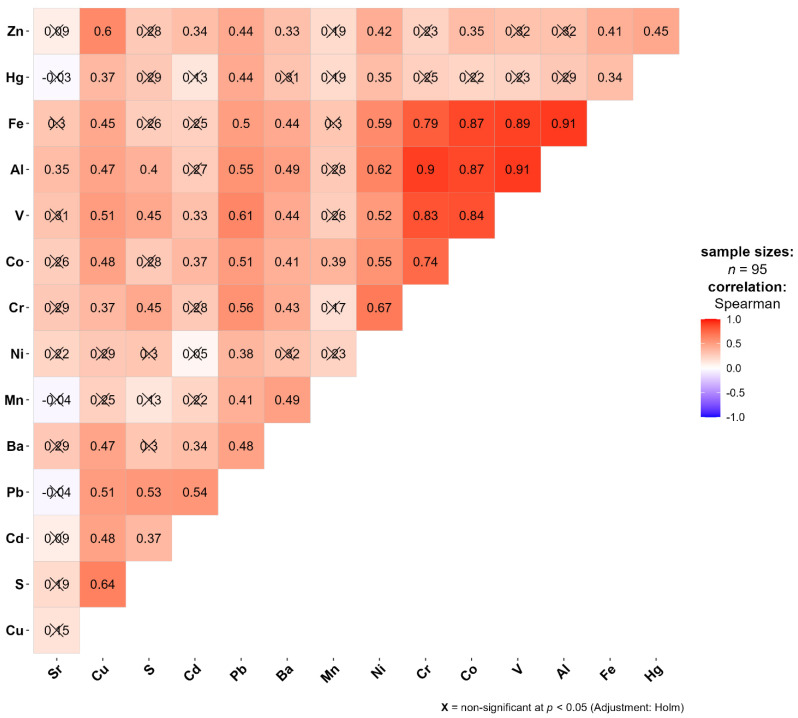
Correlation matrix between the elements of the entire initial data set. X stands for not significant.

The correlation matrix shows a very strong correlation between Cr, Co, V, Al, and Fe with correlation coefficient (r) values ranging from 0.74 to 0.91 (Figure 1). Such high correlations may be explained by the single source of provenance, and most likely it is a natural source such as soil dust particles [34].

The pronounced adsorptive affinity of vanadium towards iron oxide minerals in soil is a well-documented phenomenon that can be a significant factor influencing its distribution and concentration in the environment [35]. Other elements like Cu, Pb, and Ni also show high correlations with soil particles [36,37]. The strong correlations of Cu, Zn, Pb and Cd can be explained by a variety of sources, but vehicle emissions may play a significant role [38,39,40,41,42].

**Figure 2:**
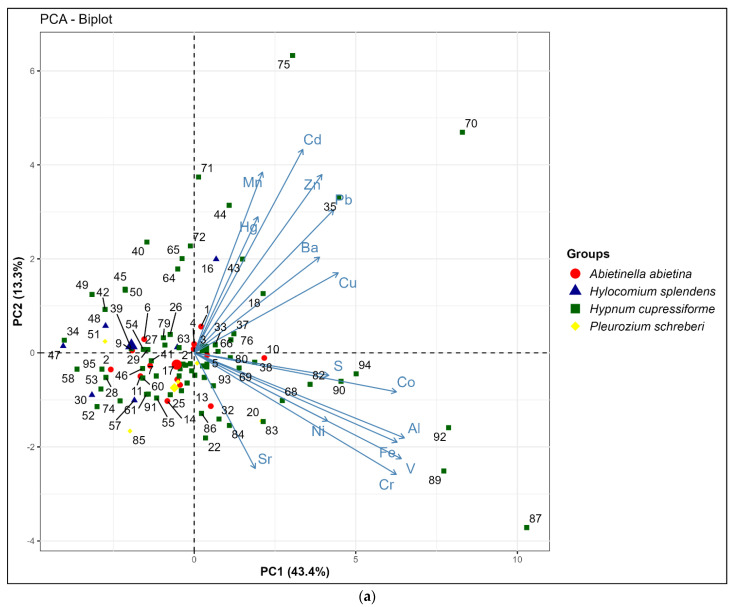

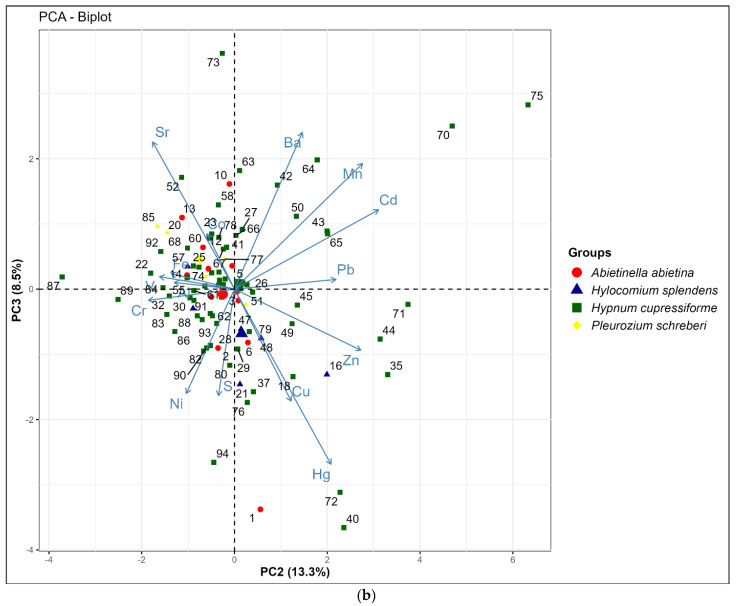
(**a**) Biplot of PC1 and PC2 denote the first two principal components explaining 56.7% of the variance in the data. (**b**) Biplot of PC2 and PC3 represent the second and third principal components explaining 21.8% of the variance in the data. Each point, distinguished by a unique combination of color and symbol, represents a sample of one of the four species. Arrows emanate from the origin, representing the variables.

In the context of Principal Component Analysis (PCA), the first principal component (PC1) accounts for 43.4% of the variance of the data (Figure 2). This PC includes elements related to geogenic origin, including Al, Co, Cr, Fe, and V. The most significant contributions come from samples No. 87, 89, 92, 94, and 90 collected in areas close to agricultural lands. It is suggested that agricultural practices on these lands, particularly during the dry seasons, contribute to the release of soil particles into the atmosphere. The open nature of these areas further aids in the dispersion of soil dust particles over a wide territory [43,44].

The second principal component (PC2) describes 13.3% variation in the data. The main contributions are made by Cd, Hg, Mn, Pb and Zn and are essentially associated with anthropogenic sources such as highways, urban areas, and industrial zones (Zestafoni ferro-alloy plant), namely the following samples: No. 70 (Kutaisi), No. 75 (Zestafoni), No. 35 (Rikoti Pass), No. 71 (Sataflia). Samples No. 70 and 71 are located near the city of Kutaisi.

The third principal component (PC3) explains 8.5% of the variation in the data (Figure 2b). The primary contributions to PC3 are made by elements Ba, Cu, Hg, Mn, Ni, S, and Sr, which can be associated with local anomalies. It must be admitted that Cu, Hg, Ni, and S have negative correlation with PC3, while Ba, Cd, Mn, and Sr have positive correlation. In the first set of cases, samples No. 1, 40, 72, 76, and 94 make significant contributions, while in the second set, samples No. 63, 64, 70, 73, and 75 stand out.

It is noteworthy to mention that sample No. 72 is located in close proximity to the port city of Poti. The elevated Cu values observed could be potentially linked to the activities at the port. Copper-based antifouling paints are widely used because they effectively slow the growth of marine organisms on boat hulls, preventing them from attaching and causing damage or slowing the boat down. However, these paints continuously release Cu into the water, leading to elevated element levels in marine basins. This problem is significantly worsened when boats are cleaned in the water, as large quantities of Cu can be washed off and released into the marine environment [45,46]. Another element that was particularly prominent at this sampling location was Hg. The elevated mercury levels detected near the port city where the Rioni River empties into the Black Sea may be attributed to several interconnected factors. Industrial activities in the region, including mining operations, may increase Hg emissions and alter natural Hg cycling processes. One significant contributing element appears to be the Mn mining activities in the town of Chiatura [47,48]. The Kvirila River, which flows past the Mn mine at Chiatura, likely transports Mn-rich sediments and runoff into the Rioni River system [49]. Manganese oxides play a crucial role in Hg speciation and transport in marine environments. They can adsorb and mobilize Hg species, potentially enhancing its bioavailability [50]. The Black Sea presents unique conditions favorable for Hg methylation and transport. Its anoxic waters extending to great depths create an environment conducive to the formation of methylmercury, the most toxic form of Hg [51]. Manganese-rich sediments could facilitate these processes. While the exact mechanisms remain complex and require further investigation, the available evidence suggests that Mn from the Chiatura mine likely contributes to elevated Hg levels in the area through enhanced transport and methylation in the Rioni River system and Black Sea. This underscores the need for comprehensive monitoring and management strategies to mitigate environmental impacts from industrial activities in this ecosystem. Additional examples about other samples from this list are provided below; otherwise, further investigation may be required.

**Figure 3:**
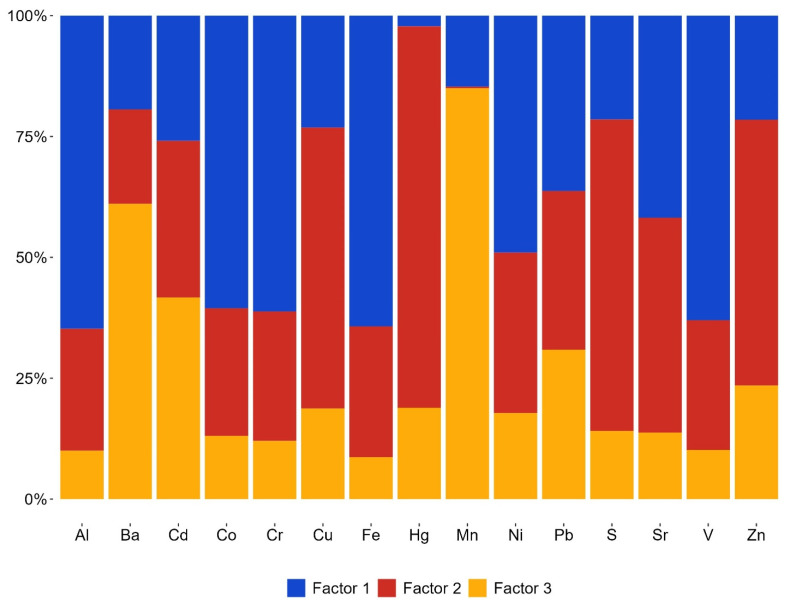
PMF analysis factor fingerprint showing the percentage contribution of three identified factors (Factor 1, Factor 2, Factor 3) across various elements measured in the moss samples.

Based on the PMF model findings the optimal solution identified three sources (Figure 3). The primary component identified by the PMF model is soil dust. This component predominantly comprises crustal elements, primarily lithophilic Al (64.8%), V (63.0%), and Cr (61.2%), and siderophilic Fe (64.3%), Co (60.5%), and Ni (49%).

The second factor primarily encompasses chalcophilic elements, specifically Hg (78.9%), S (64.4%), Cu (58.1%), and Zn (55%), which may originate from anthropogenic sources. Chalcophilic elements are those that tend to bond with sulfur (S) and can be found in sulfide minerals. These elements often come from human activities such as mining, industrial processes, and waste disposal [52,53].

The third factor characterized by elements such as Mn (85%), Ba (61.1%), and Cd (41.7%) appears to be a combination of anthropogenic and geogenic sources. Cadmium and Pb were almost equally distributed between factors in the following proportions: Cd at 25.9%, 32.4%, and 41.7% and Pb at 36.3%, 32.8%, and 30.9%, respectively. Sr exhibited equal distribution between the first and second factors, with percentages of 41.8% and 44.4%

**Figure 4:**
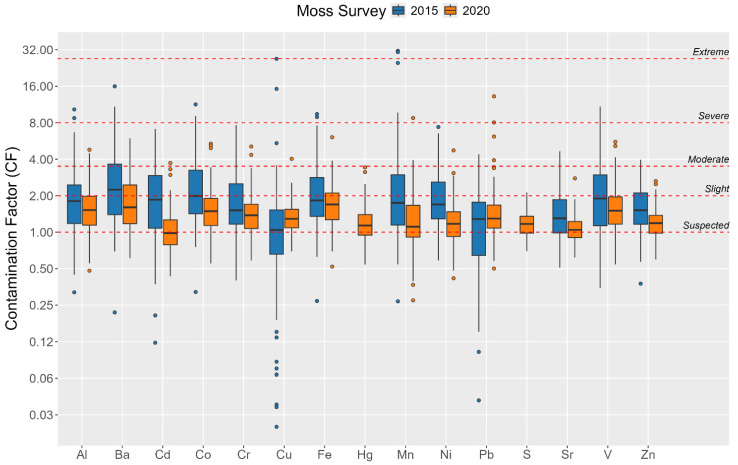
Comparison of Contamination Factors (CF) between current (95 Samples) and previous (120 Samples) moss surveys in Georgia.

Comparison of the median values of the CFs for the moss samples collected in past versus current surveys reveals a general trend of lower median values in the present survey (Figure 4). However, an exception to this pattern is observed for Cu, where the CF is notably higher. Furthermore, the distributions of Fe and Pb are found to be not significantly different (*p* > 0.05), as confirmed by the Mann–Whitney U Test.

Severe CFs must be admitted for Pb and Mn. In the case of Mn, the highest concentration was observed not far from the important industrial center Zestafoni. The major industrial facility in this area is Zestafoni Ferroalloy Plant, where raw manganese ore is transported by rail through the Kvirila valley from Chiatura. A study performed by Avkopashvili et al. [49] has revealed that in the vicinity of the manganese industrial region, particularly in the surrounding agricultural fields, Mn concentrations significantly exceed typical levels. However, this effect becomes significantly more pronounced in areas near ferroalloy plants that process ores, as opposed to locations where only mining activities occur. This is probably attributed to the application of suboptimal ore processing methods, which emit harmful pollutants into the air that then contaminate the soil through precipitation. The present study shows a similar scenario. In particular, the average concentration of Mn in mosses collected near the Chiatura mining sites (Samples No. 42,43) were approximately 2.4 times lower than in the vicinity of the ferroalloy plant (Sample No. 75).

Regrettably, our previous study did not include moss sampling in the vicinity of Zestafoni; however, it must be emphasized that the mean Mn content near the mining site is 6.5 times lower than previously reported [23]. This significant discrepancy can be explained by several factors, including variations in sampling locations, differing methodologies employed for element determination, and the acknowledged unreliability of biomonitoring using mosses as a tool for assessing atmospheric Mn deposition. The reliability of moss biomonitoring for evaluating atmospheric Mn deposition is questionable due to its dependence on environmental conditions and the sources and types of emissions. Despite atmospheric contributions, Mn concentration in moss samples may decrease under certain circumstances [54].

Severe CFs for Pb are associated with samples collected close to Kutaisi (Sample No. 70) and the vil. Lopota (Sample No. 94). If in the case of Kutaisi, a combination of several anthropogenic factors, such as transport and industry, can be assumed as the source of Pb; then, in the case of Lopota, this is most likely due to the recent construction of the Lopota Hydro Power Station (6 MW).

It is important to mention that in the research conducted by Tomczyk et al. [55] the levels of Pb found in the examined sediments were greater upstream of the hydropower facility compared to downstream locations. In our case, moss samples were collected upstream, which raises the possibility that the elevated Pb concentrations could be attributed to sediment particles contaminating the moss samples. This potential contamination source needs to be carefully considered and addressed in future studies to ensure accurate interpretation of the results.

**Figure 5:**
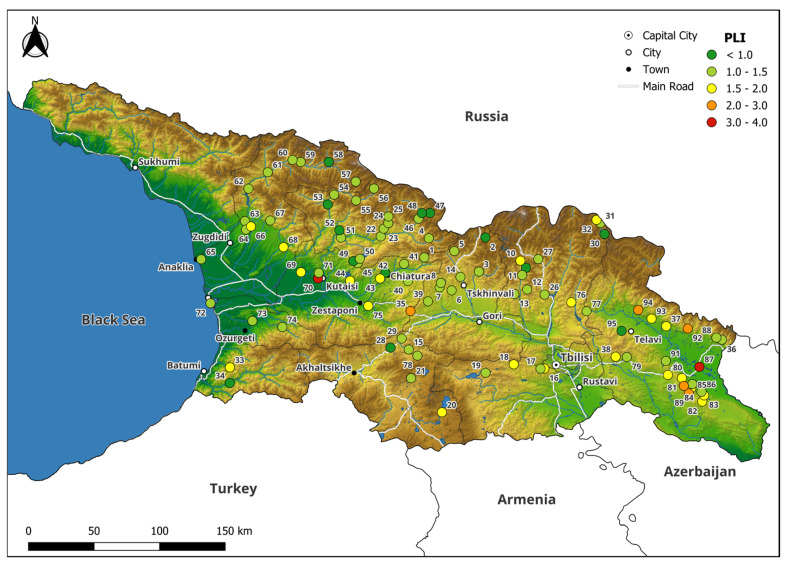
Spatial distribution of the Pollution Load Index (PLI) across all sampling locations. The PLI is represented by colored dots on the map, with different colors indicating varying levels of pollution. Each dot corresponds to a specific sampling site, numbered for reference purposes.

ArcGIS was employed to generate a spatial representation of pollution distributions, utilizing the Pollution Load Index (PLI) to show variations in pollution levels across Georgia (Figure 5).

The highest PLI was observed at sites No. 87 (Heretiskari) and No. 70 (Kutaisi) with values of 3.21 and 3.06, which indicates a pollution level from moderate to severe. Sample No. 70 is located in Kutaisi, the third-largest city in terms of population within Georgia, which also served as an important industrial center of the country. However, after the dissolution of the Soviet Union, many of the city’s manufacturing enterprises either ceased operations or significantly reduced their operations the (Auto Mechanical Plant, established in 1945, is a notable legacy of Kutaisi’s industrial past). It must be admitted that the industrial zone is located about 3 km away from the sampling site. The main industry directions are manufacturing home appliances, wood and stone processing, metal construction, and furniture and mattresses production. In the research conducted by Surmava et al. [56], the highest concentration of PM_2.5_ and PM_10_ were detected in a close vicinity to our sampling site in Kutaisi.

Sample No. 87 is located not far away from the village Heretiskari and close to the river Alazani. The main activity here is agriculture. However, this sample had the highest PLI observed, so further investigations are needed. Through analysis of satellite imagery, it became evident that samples were collected within an old riverbed, a detail that was not immediately apparent on-site. It is plausible that during periods of drought, both soil dust and river sediment, carried by wind erosion onto the moss samples, contributed to these unusually high elements contents [57].

The moderate PLI values, ranging from 2.97 to 2.22, were found in descending order within the following samples: 92, 89, 94, 35, and 90. The samples No. 92, 89, 94, and 90, which have already been mentioned above, are associated with erosion of soil particles on agriculture lands. Meanwhile, sample No. 35 is associated with a highway of international importance passing through the Rikoti Pass.

**Figure 6:**
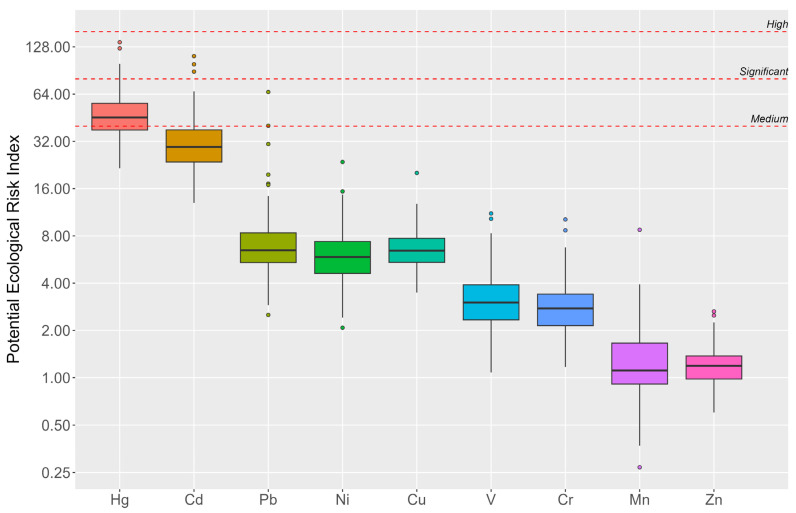
Boxplots of the Potential Ecological Risk Index (PERI) for selected elements accumulated by the mosses.

The Potential Environmental Risk Index (PERI) highlighted significant risks associated with Hg and Cd in several cases. However, Pb presented medium risks in just two samples, with all other elements showing low risks (Figure 6).

A significant PERI (112) of Cd was discovered for site No. 65, which is close to the Black Sea in Anaklia. To establish a deep-water port, efforts were underway to both deepen the water body and elevate the shoreline. This elevation was achieved by utilizing the excavated materials, but as a result of the construction process being suspended, the environmental and health hazards associated with dust emissions from these abandoned materials appeared. It is plausible that the presence of dust contributed significantly to the elevated levels of cadmium observed; therefore, further investigation is needed [58].

Significant PERI values were observed for Kutaisi and Zestafoni, with scores of 99 and 89, respectively. These high scores indicate substantial ecological concerns, which are primarily associated with industrial activities in these areas [49,56,59,60].

The highest content of Hg, at 0.113 mg/kg, was detected in sample No. 40. Approximately 5 km north of this site, near the village of Savane, a quarry extracting quartz sand is located. Industrial activities associated with mining and processing minerals can release Hg into the environment through various pathways, including air emissions and waste disposal practices. Specifically, the process of mining and crushing rock can liberate Hg that was previously bound within the mineral structure, making it available for atmospheric deposition [61]. Further investigation is needed to assess the extent of Hg contamination around the quarry and to develop strategies for monitoring and mitigating its effects on the environment and public health.

The next highest PERI, which was 125, corresponds to sample No. 35 and is linked to the Rikoti Pass. In 2023, construction of a new 27-km section was completed, including 65 bridges and 38 tunnels. It is well-established that anthropogenic Hg emissions are attributed to vehicle exhaust gases, motor fluids, and the wear of brakes and tires. Additionally, the construction and maintenance of roads contribute significantly to these emissions. Furthermore, the abrasion of paved surfaces due to vehicular traffic plays a crucial role in the release of mercury into the environment [62].

The Ecological Risk Index (ERI) indicated low risk across most sampling locations, with only 16 samples showing a moderate level of ecological risk, ranging from 150.5 to 254.7. The highest ERI was identified in samples No. 70 (Kutaisi) and No. 35 (Rikoti Pass); one is associated with an industrial zone and the other with the construction of a new highway connecting the eastern and western parts of the country.

## 3. Materials and Methods

### 3.1. Study Area

Georgia is a transcontinental country situated in Eastern Europe and Western Asia in the Caucasus region, with access to the Black Sea. It shares borders with Russia to the north and northeast, Turkey to the southwest, Armenia to the south, and Azerbaijan to the southeast. The country encompasses a total area of 69,700 km^2^ and has a population of around 3.7 million people. Georgia’s landscape is characterized by its mountainous terrain, with the Greater Caucasus range defining much of its northern boundary and creating natural barriers. This mountainous nature leads to a variety of ecosystems and contributes to the country’s biodiversity. The Caucasus Mountain range significantly influences Georgia’s climate, leading to variations in climatic elements like air temperature and precipitation, not by latitude but by hypsometrical zonality [63].

The complex topography, meridional orientation of the Caucasus, and elevated terrain of southern Georgia, along with the influence of adjacent seas, significantly alter atmospheric circulation patterns. This unique combination of geographical features results in a diverse climate within a relatively compact area. Consequently, dry steppes with annual precipitation below 400 mm coexist alongside regions with extremely high humidity, receiving over 4000 mm of precipitation annually. Precipitation varies significantly across different regions, ranging from 1600–1900 mm in the coastal zones of Kolkheti to 400–1800 mm in eastern Georgia. The Meskheti Range’s seaward slopes receive the highest rainfall, averaging 4500 mm annually at Mt. Mtirala, while the southeastern part of eastern Georgia experiences the lowest, with less than 400 mm. The wind regime, shaped by the seasonal pressure distribution over Eurasia and the Black and Caspian Seas, along with Georgia’s complex topography, contributes to these variations. Western Georgia is dominated by eastern terrestrial winds in winter and western marine winds in summer, with local winds like breezes and foehns further influencing the climate. The Likhi Range acts as a climatic divide, affecting wind patterns and contributing to the diverse climatic conditions across Georgia [63].

### 3.2. Moss Sampling

**Figure 7:**
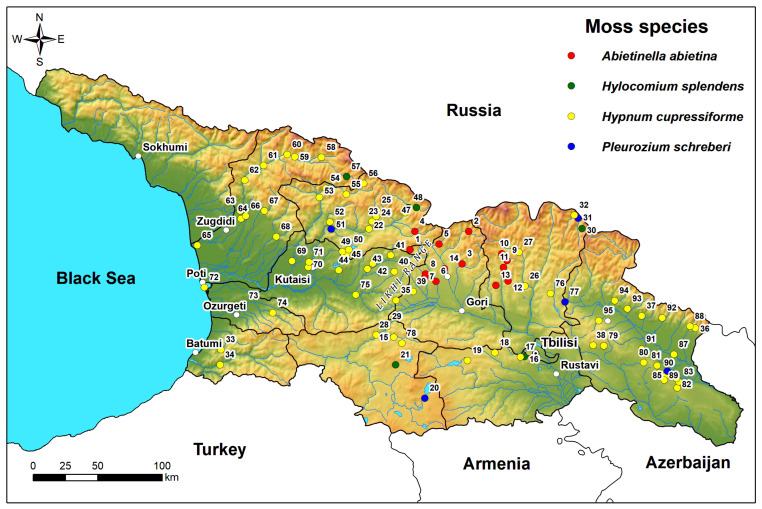
Map of sampling locations with color-coded markers to indicate moss species collected. Red dots indicate *Abietinella abietina*, green—*Hylocomium splendens*, yellow—*Hypnum cupressiforme*, and blue—*Pleurozium schreberi*.

During a 2019–2023 moss survey in Georgia, 95 moss samples were collected, covering most of the country’s territory. Sampling locations are visualized on the map depicted in Figure 7. Mostly *Hypnum cupressiforme* Hedw. (n = 70) was sampled, but in case of its absence *Abietinella abietina* (Hedw.) M. Fleisch. (n = 14), *Pleurozium schreberi* (Brid.) Mitt. (n = 5), and *Hylocomium splendens* (Hedw.) Schimp. (n = 6) were collected. The elevation of sampling locations ranged from 2 to 2123 m above sea level. It should be noted that *H. cupressiforme* was not found at altitudes above 1800 m.a.s.l. Utilizing several traditionally used moss species in a moss survey to cover the whole territory of the country is a methodology that has been adopted by numerous countries [15,20,64]. The sampling was conducted by ICP Vegetation—the moss survey protocol’s standard methodology [31].

### 3.3. Sample Preparation and Analysis

Analysis was conducted solely on the green shoots of mosses, which typically represents growth over the last 2–3 years. To reduce the risk of sample contamination disposable polyethylene gloves and plastic tweezers were employed during the cleaning process to remove extraneous materials. Post-cleaning, the samples were dried until they reached a constant weight, specifically at 105 °C for a duration of 48 h. The samples underwent thorough pulverization to achieve a uniform consistency using a Fritsch Pulverisette 6 planetary mill equipped with an agate grinding bowl and balls. The samples were subjected to dissolution processes; namely, approximately 500 mg of moss was placed into a Teflon vessel for digestion, utilizing 5 mL of concentrated nitric acid (HNO_3_) and 2 mL of hydrogen peroxide (H_2_O_2_), at a temperature of 180 °C in a microwave digestion system (Mars; CEM, Matthews, NC, USA). Following digestion, the resultant solutions were carefully transferred to 50 mL calibrated flasks and diluted to the mark with bi-distilled water. The chemicals employed throughout this study were of analytical grade, including 69% nitric acid (Merck, Darmstadt, DE, Germany) and 30% hydrogen peroxide (Merck, Darmstadt, DE, Germany), alongside bi-distilled water.

The concentrations of 15 elements, including Al, Ba, Cd, Cr, Co, Cu, Pb, Fe, Mn, Ni, S, Sr, V, and Zn, were determined using inductively coupled plasma atomic emission spectroscopy (ICP-AES, PlasmaQuant PQ 9000 Elite, Analitik Jena, Jena, Germany). The content of Hg was determined using a direct mercury analyzer (DMA-80 evo, Milestone Srl, Sorisole, BG, Italy). Mercury determination did not require sample dissolution processes.

The quality control of ICP-AES results using certified reference materials (CRMs) such as M2 (*Pleurozium schreberi*) and INCT-OBTL-5 (Oriental Basma Tobacco Leaves), demonstrated recovery rates for the majority of the measured elements to be within acceptable ranges. Specifically, the recovery rates were 90–113% for M2, and 79–100% for INCT-OBTL-5 (Table S2).

### 3.4. Correlations Analysis

Correlation analysis was used to evaluate the strength and direction of relationship between element concentrations. Due to the data’s non-normal distribution, Spearman’s correlation was performed using the ggstatsplot package in R, including the application of the Holm correction for multiple comparisons [65]. The Holm adjustment, also known as the Holm–Bonferroni method, is used in statistical analysis to control the probability of making one or more Type I errors (false positives).

### 3.5. Mann–Whitney U Test

For the purpose of comparing the outcomes of previous and current moss surveys, a nonparametric statistical test known as the Mann–Whitney U test was employed. This methodological approach was chosen due to the absence of strict requirements for data distribution.

The calculations for the Mann–Whitney U test were performed using the R programming environment. Specifically, the wilcox.test() function from R’s built-in stats package was utilized to conduct the analysis.

### 3.6. Principal Component Analysis

Principal Component Analysis (PCA) is a widely utilized technique in biomonitoring and other fields for dimension reduction and feature extraction. PCA operates by geometrically projecting the data onto fewer dimensions known as principal components (PCs), which summarize the data’s features. The first principal component is selected to maximize the variance of the projected data, capturing the most significant pattern. Subsequent PCs are chosen to be orthogonal (uncorrelated) to the preceding ones, ensuring that each PC captures unique variance in the data. This orthogonal property limits the maximum number of PCs to the lesser of the number of samples or features. PCA was performed using the FactoMineR package in R, and the results were subsequently visualized with the help of the factoextra package [66,67].

### 3.7. Positive Matrix Factorization Analysis

The Positive Matrix Factorization (PMF 5.0) is a mathematical receptor model developed by the Environmental Protection Agency (EPA). The PMF model simplifies complex analytical data sets by reducing the large number of variables to combinations of species called source types and source contributions. This simplification process involves assigning weights to each data point, taking into account both the concentration of the sample and its uncertainty. Source types are identified by comparing them to measured profiles, while source contributions are used to determine how much each source contributed to a sample. PMF is particularly useful for discovering hidden patterns [68].

### 3.8. Background Calculation

Identifying uncontaminated sites to establish baseline levels for elements is challenging due to widespread contamination. The method described by Krakovska involves sorting element concentrations from lowest to highest, assigning an order to each sampling point for each element, summing these orders, and then using the mean concentration of the first 5% of points as the background value [69].

In our case, we utilized 10% of the dataset due to its limited size. Outliers were tested using a Grubbs’ test at a significance level of 0.05, for ensuring that the analysis is not skewed by extreme values that do not represent uncontaminated sites. To enhance the reliability of our findings, bootstrapping was employed to establish the upper limit of the 95% confidence interval for the mean value. This method aims to provide a more accurate representation of background levels by incorporating the natural variability and distribution of element concentrations across sampling points.

### 3.9. The Contamination Factor

The calculation of the Contamination Factor (CF) serves as a measure for evaluating the level of contamination by specific elements in a given area. The CF is determined by dividing the concentration of a specific element found in a moss sample by its background concentration, as shown in Equation (1).

(1) $$CF=\frac{C_{m}}{C_{b}},$$

In this mathematical expression, *C_m_* denotes the concentration of a chosen element, whereas *C_b_* signifies the background level of the identical substance. The degree of contamination is categorized based on the value of the CF [70]. The classification is presented in Table 3.

### 3.10. The Pollution Load Index

The Pollution Load Index (PLI) serves as a comprehensive measure for evaluating the extent of pollution within a given sample. It is determined through the calculation of the nth root of the product of Contamination Factors (CFs). The formula is structured as follows:

(2) $$PLI=\;\sqrt[{9}]{CF_{Al}\times CF_{Cd}\times CF_{Cr}\times CF_{Cu}\times CF_{Fe}\times CF_{Ni}\times CF_{Pb\;}\times CF_{V\;}\times CF_{Zn\;}},$$

The scoring system for the PLI ranges from 1 to 6, where 0 indicates no pollution, 1 signifies minimal to moderate pollution, 2—moderate pollution levels, 3—moderate to severe pollution, 4—severe pollution, 5—severe to extremely high pollution, 6—extremely high pollution [71].

### 3.11. The Potential Ecological Risk Index

The Potential Ecological Risk Index (PERI) serves as a diagnostic tool to assess the ecological risks posed by chemical elements found in the moss samples. This index integrates both the contamination factor (CF) and the toxic-response factor (TRF) for each element. The PERI is calculated using the following formula:

(3) $$PE{RI}_{i}=\;CF_{i}\times \;TRF_{i}$$

where *CF_i_* is the contamination factor for single element, and *TRF_i_* is the toxic-response factor for the same element. Different elements have assigned TRF values based on their known toxicities, such as 1 for Mn and Zn, 2 for C, and V, 5 for Cu, Pb, and Ni, 30 for Cd, and 40 for Hg [72].

(4) $$ERI=\sum_{n=1\;}^{n}{\left(PE{RI}_{i}\right)}_{n},$$

The Potential Ecological Risk Index (PERI) for each element is classified into five distinct levels, as follows: a PERI less than 40 indicates a low risk level; values ranging from 40 to 80 suggest a medium risk; those between 80 and 160 denote a significant risk; those within the range of 160 to 320 signify a high risk; and finally, a PERI greater than 320 represents an extreme level [73].

Conversely, the Ecological Risk Index (ERI) is divided into four categories: low risk, indicated by an ERI value less than 150; moderate risk, applicable for values between 150 and 300 (inclusive); considerable risk, relevant for values between 300 and 600 (inclusive); and high risk, applicable for ERI values greater than or equal to 600. These categorizations serve to pinpoint regions exhibiting substantial environmental issues stemming from metal contamination [74].

## 4. Conclusions

This comprehensive study on air deposition of major and trace elements in Georgia provides valuable insights into the current state of environmental contamination across the country. Our findings reveal a general trend of decreasing median values for most elements compared to a previous survey performed in Georgia, with notable exceptions being unchanged levels of Cd and Zn, and increased Cu content.

The analysis identified strong correlations among naturally occurring elements like Cr, Co, V, Al, and Fe, likely originating from soil dust. Principal Component Analysis revealed three primary sources of elemental distribution: geogenic origins primarily from agricultural areas, anthropogenic sources associated with highways and industrial zones, and local anomalies with mixed correlations.

Our research highlights severe contamination levels for Pb and Mn, particularly near industrial centers such as the Zestafoni Ferroalloy Plant. Manganese concentrations were significantly higher near ferroalloy plants compared to mining sites alone, while Pb contamination was linked to anthropogenic factors around Kutaisi and the Lopota Hydro Power Station. The Ecological Risk Index revealed low to moderate risks across most sites, with notable risks in industrial and construction-related areas.

These findings emphasize the need for continued monitoring and mitigation strategies to address potential ecological risks associated with deposition of trace elements in Georgia. This study serves as a crucial baseline for future assessments and policy development aimed at protecting Georgia’s environment and public health. Future research should focus on expanding sampling sites to encompass remote regions and sensitive ecosystems throughout the country, providing a more comprehensive understanding of elemental distribution and its environmental impacts.

## Figures and Tables

**Table 1:** Descriptive statistics of element content (in mg/kg) in 95 moss samples collected in Georgia in the period of 2019–2023.

Element	Range	Mean ± st.dev	Median ± MAD	CV%	Q1	Q3	Percentile 90	Background
Al	674–6708	2361 ± 1106	2131 ± 616	47%	1601	2775	3862	1400
Ba	10.9–105.7	33 ± 17.5	28.6 ± 10.2	53%	20.9	43.6	54.9	17.8
Cd	0.06–0.49	0.15 ± 0.07	0.13 ± 0.03	49%	0.10	0.17	0.22	0.13
Co	0.33–3.17	0.99 ± 0.5	0.88 ± 0.22	51%	0.67	1.12	1.44	0.59
Cr	1.57–13.68	4.09 ± 1.9	3.71 ± 0.86	47%	2.88	4.58	6.02	2.69
Cu	3.81–22.08	7.56 ± 2.48	7.06 ± 1.26	33%	5.95	8.47	10.5	5.49
Fe	561–6548	1961 ± 890	1826 ± 457	45%	1370	2268	3113	1079
Hg	0.018–0.113	0.041 ± 0.016	0.037 ± 0.007	38%	0.031	0.046	0.054	0.033
Mn	26.2–835.7	141.2 ± 104.7	105.9 ± 36.7	74%	86.8	159	267	95.5
Ni	1.57–17.82	4.95 ± 2.36	4.41 ± 1.02	48%	3.48	5.55	7.77	3.77
Pb	1.61–42.32	5.38 ± 5.05	4.16 ± 1.05	94%	3.47	5.36	8.08	3.21
S	746–2281	1294 ± 331	1252 ± 199	26%	1054	1449	1703	1071
Sr	21.4–96.7	37.7 ± 11.6	36.2 ± 6.0	31%	31.3	42.6	50.2	34.8
V	1.91–19.62	5.86 ± 2.93	5.32 ± 1.41	50%	4.12	6.90	9.21	3.53
Zn	14.17–62.81	29.45 ± 8.92	28.25 ± 4.87	30%	23.3	32.7	41.0	23.8

**Table 2:** Comparison of median moss concentrations from the current study with the previous study [23] values published by Hristozova et al. [25], Tepanosyan et al. [26], and Nurkassimova et al. [27].

Element	Georgia, 2019–2023		Georgia, 2014–2017		Bulgaria, 2015–2016		Armenia, 2016–2017		South Kazakhstan, 2019	
	(n = 95)		(n = 120)		(n = 115)		(n = 65)		(n = 32)	
	Median	Range	Median	Range	Median	Range	Median	Range	Median	Range
Al	2131	674–6708	4295	759–24,500	2310	569–10,900	–	–	10,550	33.8–40,300
Ba	28.6	10.9–106	51.3	4.98–365	46	14.2–309	99.8	21.9–313	146.5	36.6–439
Cd	0.13	0.06–0.49	0.15	0.01–0.58	0.1	0.02–1.56	–	–	0.12	0.07–0.73
Co	0.88	0.33–3.17	1.43	0.23–8.12	0.59	0.197–3.29	3.7	0.9–15.4	3.06	0.66–10.1
Cr	3.71	1.57–13.68	7.75	2.04–39.00	2.73	0.219–25	22.3	5.7–243	16.75	2.6–60.7
Cu	7.06	3.81–22.08	5.54	0.13–143.07	7.36	3.2–46.88	14.2	5.3–212	3.95	1.59–15.3
Fe	1826	561–6548	2725	404–14,100	1190	376–7240	7380	1840–29,100	7940	1580–25,900
Hg	0.037	0.018–0.113	–	–	–	–	–	–	–	–
Mn	106	26.2–835.7	141	21.7–2530.0	180	39–551	225	61.8–686	210.5	70.5–1260
Ni	4.41	1.57–17.82	5.56	1.92–24.20	2.1	0.45–13.5	–	–	7.38	0.2–36.6
Pb	4.16	1.61–42.32	5.57	0.18–19.1	10.7	3.72–102.8	6.1	2.5–34.3	3.31	1.26–43.46
S	1252	746–2281	–	–	–	–	–	–	–	–
Sr	36.3	21.4–96.7	43.9	17.2–157.0	25	11.3–122	82.5	37–286	86.25	29.3–521
V	5.32	1.91–19.62	9.4	1.71–54.00	3.89	1.3–22.7	24.4	6.5–84.0	16.65	0.47–64
Zn	28.25	14.17–62.81	28.9	7.15–75.20	28	9–101	42	22–149	65.35	22–177

**Table 3:** Classification of Contamination Factor (CF).

Contamination Factor (CF) Value	Degree of Contamination
CF < 1	No Contamination
1 < CF < 2	Suspected
2 < CF < 3.5	Slight
3.5 < CF < 8	Moderate
8 < CF < 27	Severe
CF > 27	Extreme

## Data Availability

All data are presented in the manuscript.

## Supplementary Materials

The following supporting information can be downloaded at: https://www.mdpi.com/article/10.3390/plants13233298/s1, Table S1: Descriptive statistics and Table S2: Quality control.
