# Supplementary material for: Major and Trace Airborne Elements and Ecological Risk Assessment: Georgia Moss Survey 2019–2023
Source: Plants (Basel). 2024 Nov 23;13(23):3298. doi: 10.3390/plants13233298 (PMC11644778; doi:10.3390/plants13233298)
Supplement: Supplementary file 1 [file plants-13-03298-s001.zip › plants-3268663-SI.pdf]

Table S1. Descriptive statistics for each moss species

Hypnum Cupressiforme **(n=70)**						
**Element**	**Range**	**Mean ± st.dev**	**Median ± MAD**	**CV%**	**Q1**	**Q3**
Al	776 - 6708	2483 ± 1182	2288 ± 564	48%	1685	2829
Ba	10.9 - 106	33.5 ± 18.3	28.9 ± 9.8	54%	21	42
Cd	0.06 - 0.49	0.16 ± 0.08	0.13 ± 0.03	49%	0.11	0.17
Co	0.39 - 3.17	1.06 ± 0.55	0.96 ± 0.26	52%	0.69	1.19
Cr	1.57 - 13.68	4.3 ± 2.09	3.88 ± 0.98	49%	2.93	4.91
Cu	4.54 - 22.08	7.83 ± 2.69	7.29 ± 1.47	34%	6.16	8.84
Fe	816 - 6548	2007 ± 950	1898 ± 535	47%	1369	2426
Hg	0.018 - 0.113	0.041 ± 0.017	0.038 ± 0.008	41%	0.031	0.047
Mn	26.2 - 835.7	155.8 ± 115.3	132.4 ± 45.2	74%	88	176
Ni	1.57 - 11.57	4.75 ± 1.92	4.4 ± 1	40%	3.52	5.45
Pb	1.61 - 42.32	6.09 ± 5.71	4.78 ± 1.11	94%	3.88	5.91
S	746 - 2281	1350 ± 348	1306 ± 216	26%	1108	1527
Sr	21.44 - 96.68	37.45 ± 11.87	36.2 ± 5.3	32%	31.5	41.5
V	1.94 - 19.62	6.27 ± 3.21	5.54 ± 1.52	51%	4.23	7.36
Zn	14.17 - 62.81	29.09 ± 9.01	27.46 ± 4.42	31%	23.5	32.0

Hylocomium splendens **(n=6)**						
**Element**	**Range**	**Mean ± st.dev**	**Median ± MAD**	**CV%**	**Q1**	**Q3**
Al	674 - 1979	1487 ± 562	1680 ± 294	38%	1077	1942
Ba	11.7 - 24	18.2 ± 5.1	18.9 ± 4.4	28%	14	22
Cd	0.07 - 0.18	0.12 ± 0.04	0.11 ± 0.03	34%	0.09	0.14
Co	0.33 - 1.03	0.68 ± 0.27	0.73 ± 0.2	40%	0.46	0.83
Cr	1.59 - 3.83	2.86 ± 0.94	3.18 ± 0.5	33%	2.10	3.52
Cu	3.81 - 10.32	6.73 ± 2.29	6.42 ± 1.39	34%	5.44	7.84
Fe	561 - 2129	1330 ± 624	1363 ± 534	47%	835	1768
Hg	0.028 - 0.07	0.044 ± 0.015	0.04 ± 0.009	35%	0.034	0.051
Mn	35.1 - 228.9	110.5 ± 70.6	105.2 ± 46.2	64%	59	135
Ni	1.82 - 7.31	4.24 ± 2.01	4.43 ± 1.45	47%	2.79	5.00
Pb	1.86 - 5.42	3.06 ± 1.3	2.97 ± 0.68	42%	2.13	3.23
S	762 - 1581	1191 ± 298	1239 ± 223	25%	1005	1361
Sr	25.53 - 41.12	31.5 ± 6.34	29.88 ± 4.3	20%	26.4	35.5
V	1.91 - 5.6	3.8 ± 1.54	3.93 ± 1.28	41%	2.57	4.96
Zn	19.38 - 45.63	26.76 ± 9.96	23.27 ± 3.75	37%	20.2	28.2

Pleurozium schreberi (n=5)						
**Element**	**Range**	**Mean ± st.dev**	**Median ± MAD**	**CV%**	**Q1**	**Q3**
Al	1333 - 4231	2500 ± 1108	2577 ± 810	44%	1767	2592
Ba	13.2 - 50	31.6 ± 15.3	29.5 ± 14.2	49%	21	44
Cd	0.11 - 0.2	0.15 ± 0.04	0.15 ± 0.04	28%	0.11	0.17
Co	0.59 - 1.43	0.91 ± 0.35	0.83 ± 0.24	39%	0.61	1.08
Cr	2.88 - 5.67	4.22 ± 1.09	4.1 ± 0.74	26%	3.58	4.84
Cu	4.41 - 8.48	6.28 ± 1.61	5.77 ± 1.36	26%	5.42	7.30
Fe	1132 - 3545	1912 ± 965	1804 ± 549	50%	1255	1826
Hg	0.025 - 0.053	0.034 ± 0.012	0.032 ± 0.007	34%	0.025	0.036
Mn	37.7 - 136.9	81.4 ± 39.8	91.4 ± 43.2	49%	48	93
Ni	3.11 - 8.26	5.06 ± 2.08	4.75 ± 1.31	41%	3.44	5.75
Pb	2.83 - 4.04	3.18 ± 0.49	3.02 ± 0.08	15%	2.94	3.05
S	776 - 1548	1128 ± 304	1136 ± 227	27%	909	1269
Sr	22.06 - 57.98	43.25 ± 13.22	47.13 ± 5.3	31%	42	47
V	3.33 - 6.99	4.89 ± 1.33	4.86 ± 0.48	27%	4.38	4.88
Zn	18.31 - 29.16	26.29 ± 4.51	28.25 ± 0.91	17%	27	28

Abietinella abietina (n=14)						
**Element**	**Range**	**Mean ± st.dev**	**Median ± MAD**	**CV%**	**Q1**	**Q3**
Al	1050 - 3117	2078 ± 637	2131 ± 508	31%	1499	2510
Ba	14.4 - 63	37.6 ± 15.8	33.2 ± 15.1	42%	27	51
Cd	0.07 - 0.18	0.1 ± 0.03	0.1 ± 0.01	26%	0.09	0.11
Co	0.44 - 1.12	0.8 ± 0.18	0.83 ± 0.1	22%	0.73	0.92
Cr	2.1 - 5.29	3.51 ± 0.92	3.51 ± 0.74	26%	2.77	4.16
Cu	5.13 - 10.4	7.06 ± 1.31	6.68 ± 0.53	19%	6.34	7.31
Fe	1056 - 3036	2017 ± 547	1987 ± 287	27%	1723	2264
Hg	0.031 - 0.051	0.038 ± 0.005	0.038 ± 0.002	13%	0.035	0.040
Mn	54.2 - 225.8	103.3 ± 42.7	95.8 ± 15.6	41%	87	114
Ni	2.93 - 17.82	6.22 ± 3.97	4.99 ± 1.59	64%	3.48	7.73
Pb	2.76 - 5.31	3.62 ± 0.61	3.62 ± 0.13	17%	3.55	3.76
S	869 - 1426	1117 ± 147	1119 ± 82	13%	1016	1181
Sr	25.85 - 65.01	39.65 ± 10.69	36.59 ± 6.81	27%	33	46
V	2.49 - 6.97	5.02 ± 1.39	5.21 ± 0.98	28%	3.92	6.12
Zn	22.39 - 53.54	33.55 ± 8.61	33.6 ± 5.24	26%	28	39

Table S2. Quality Control

Elements	M2			OBTL-5			LOD, mg/kg
	Experimental value, mg/kg	Cetified value, mg/kg	Recovery, %	Experimental value, mg/kg	Cetified value, mg/kg	Recovery, %	
Al	163.51	175	93	1840.8	1981	93	0.4965
Ba	17.23	17.6	98	64.8	67.4	96	0.0041
Cd	0.45	0.454	98	2.3	2.64	86	0.0001
Co	0.89	0.9	99	0.9	0.98	88	0.0004
Cr	0.93	0.92	101	-	-	-	0.0001
Cu	66.75	68.1	98	9.6	10.1	95	0.0005
Fe	255.95	245	104	1496.1	1491	100	1.024
*Hg				0.0199	0.0209	95	0.003 ng
Mn	322.03	357	90	161.5	180	90	0.1136
Ni	14.11	14.8	95	6.7	8.5	79	0.0001
Pb	6.56	5.86	112	1.8	2.01	91	0.001
S	1069.83	995	108	4256.5	4550	94	0.2215
Sr	-	-	-	97.6	105	93	0.0034
V	1.37	1.21	113	3.5	4.12	85	0.0014
Zn	35.80	35.2	102	49.8	52.4	95	0.0009

* Mercury was determined using DMA-80 evo Wide Range, detection limit is given in nanograms.
